# Sex-related differences in retinal function in Wistar rats: implications for toxicity and safety studies

**DOI:** 10.3389/ftox.2023.1176665

**Published:** 2023-05-23

**Authors:** Cheryl Tyszkiewicz, Seo-Kyoung Hwang, Balasubramanian Manickam, Ben Jakubczak, Karen M. Walters, Michael W. Bolt, Rosemary Santos, Chang-Ning Liu

**Affiliations:** ^1^ Comparative Medicine, Pfizer, Groton, CT, United States; ^2^ Drug Safety Research and Development, Pfizer, Groton, CT, United States; ^3^ Drug Safety Research and Development, Pfizer, Cambridge, Massachusetts, United States

**Keywords:** retinal function, toxicity, Wistar Han, sex, electroretinography (ERG)

## Abstract

**Introduction:** Wistar Han rats are a preferred strain of rodents for general toxicology and safety pharmacology studies in drug development. In some of these studies, visual functional tests that assess for retinal toxicity are included as an additional endpoint. Although the influence of gender on human retinal function has been documented for more than 6 decades, preclinically it is still uncertain if there are differences in retinal function between naïve male and female Wistar Han rats.

**Methods:** In this study, sex-related differences in the retinal function were quantified by analyzing electroretinography (ERG) in 7-9-week-old (*n* = 52 males and 51 females) and 21–23-week-old Wistar Han rats (*n* = 48 males and 51 females). Optokinetic tracking response, brainstem auditory evoked potential, ultrasonic vocalization and histology were tested and evaluated in a subset of animals to investigate the potential compensation mechanisms of spontaneous blindness.

**Results/Discussion:** Absence of scotopic and photopic ERG responses was found in 13% of 7-9-week-old (7/52) and 19% of 21–23-week-old males (9/48), but none of female rats (0/51). The averaged amplitudes of rod- and cone-mediated ERG *b*-wave responses obtained from males were significantly smaller than the amplitudes of the same responses from age-matched females (−43% and −26%, respectively) at 7–9 weeks of age. There was no difference in the retinal and brain morphology, brainstem auditory responses, or ultrasonic vocalizations between the animals with normal and abnormal ERGs at 21–23 weeks of age. In summary, male Wistar Han rats had altered retinal responses, including a complete lack of responses to test flash stimuli (i.e., blindness), when compared with female rats at 7–9 and 21–23 weeks of age. Therefore, sex differences should be considered when using Wistar Han rats in toxicity and safety pharmacology studies with regards to data interpretation of retinal functional assessments.

## 1 Introduction

Due to their longevity, small body size, slow growth rate, and low incidence of spontaneous tumors, Wistar Han (WH) rats are currently one of the most used strains in biomedical research (Weber et al., 2011; Gauvin et al., 2019). This strain of rat has also been recommended for use in toxicological testing in drug development in the United States (Son et al., 2010; Gauvin et al., 2019) and Europe (Gauvin et al., 2019). Sometimes visual functional tests, e.g., electroretinography (ERG) or visual discrimination behavioral tests, are included as an add-on endpoint for assessing potential retinal toxicity of new molecules (Rosolen et al., 2005; Brock et al., 2013). Ophthalmologic and histopathologic examinations have shown a higher incidence of corneal opacities and mineralization in WH rats compared with Sprague-Dawley rats (Hayakawa et al., 2013). Spontaneous microscopic lesions have also recently been reported in retinas in this strain with 5.0%–45.7% of rats examined displaying retinal degeneration and retinal rosettes/folds (Cloup et al., 2021). In previous pilot studies, as many as 11%–12% of adult male WH rats were identified as having virtually no ERG responses to a series of test light flashes, indicating a loss or decrease in visual function. Although these animals behave normally, as observed during cage-side observations, and had no findings with standard eye examinations, some of them were found to be blind based on our ERG assessments. In pharmacology or neurological studies, rats with significant photoreceptor loss (O'Steen et al., 1995) and rats with reduced visual acuity (Prusky et al., 2000) are all impaired in the Morris water task experiments, compromising the interpretation of experimental data that are dependent on visual function. It is also essential for toxicologists to be familiar with spontaneous ocular morphological and functional alternations that may occur in WH rats used in safety assessment studies. Although the visual responses at retina (Heiduschka and Schraermeyer, 2008), brain (Thomas et al., 2005), visual acuity threshold (Prusky et al., 2002) and susceptibility to light damage (De Vera Mudry et al., 2013) have been compared between pigmented and albino rats, no comparative study has quantified the visual or retinal function of WH rats in large groups of male and female WH rats.

Visual impairment or blindness can alter sensory, memory, social, and survival behavior through various compensatory mechanisms. Since the 1980s, evidence has accumulated showing that blind individuals can have better hearing than those with normal vision, due to intramodal plasticity in the cortex and subcortical auditory structures (Niemeyer and Starlinger, 1981; Liotti et al., 1998; Bavelier and Neville, 2002). Alterations in auditory brainstem responses have also been observed in blind adults (Jafari and Malayeri, 2014) and children (Jafari and Malayeri, 2016). However, these forms of intramodal compensation have not been documented in blind or vision-impaired rodents.

To fill the knowledge gaps, the current study screened male and female adult WH rats using regular ophthalmic examinations and ERG. Additionally, optokinetic response tracking (OKR), brainstem auditory evoked potential (BAEP), and ultrasonic vocalization (USV) were performed or recorded to compare the potential differences between normal-sighted and blind animals. The resulting structural plasticity in the retina, visual, and auditory pathways in the brain was also examined using conventional histology methods.

## 2 Materials and methods

### 2.1 Animals

All activities involving animals conformed to the guidelines established by the Association for Research in Vision and Ophthalmology (ARVO) statement for the Use of Animals in Ophthalmic and Vision Research, and the animal use protocol was approved by the Pfizer Institutional Animal Care and Use Committee (IACUC). Adult male and female WH rats (Crl:WI [Han], Charles River Laboratories, Raleigh, NC) were obtained at an age of approximately 6–10 weeks of age. The animals were group-housed (2–3/cage) in Techniplast cages with Enrich-n’Pure bedding (The Andersons Inc., Maumee, OH) with a room temperature of 20°C–26°C and humidity of 30–70 %, under a 12 h:12 h light-dark cycle. They were provided with *ad libitum* reverse osmosis purified water and a regular irradiated Teklad Global Rodent Diet (Envigo, 2916C). ERG and OKR tests were performed on all animals between 8:00 a.m. and 3:00 p.m. Three cohorts of WH rats were ordered (see Supplementary Table S1) and assigned to four groups in this study as summarized in Table 1. Group 1 and 3 consisted of 2 separate sets of male rats, one 7–9 weeks of age (*n* = 52) and the other 21–23 weeks of age (*n* = 48). Group 2 and 4 consisted of the same set of female rats (*n* = 51) evaluated at 7–9 weeks of age and then again at 21–23 weeks of age.

**TABLE 1:** Incidence of blindness in Wistar Han rats.

Group	Age (week)	Sex	# Of animals with abnormal ERG	Total # of animals tested	Incidence of blindness (%)	*p*-Value compared with group 1 animals
1	7–9	Male	7	52	13	N/A
2		Female	0	51	0	0.0126
3	21–23	Male	9	48	19	0.5877
4^a^		Female	0	51	0	0.0126

^a^ The same animals as group 2 tested at 21–23 weeks of age.

### 2.2 Ophthalmologic examination

A standard qualitative ophthalmic examination was conducted either prior to ERG testing for group 2 animals (females) at 7–9 weeks of age or after the ERG assessment for groups 1 and 3 animals (males) at 7–9 weeks of age and 21–23 weeks of age, respectively. The visible ocular and adnexal anatomy were evaluated. Mydriacyl (1.0% tropicamide, Akorn Operating Company LLC, Lake Forest, IL) was applied topically to each eye to assist in the examination. In ambient lighting, indirect ophthalmoscopy was utilized to examine the retina, optic disc, and blood vessels, and a handheld slit lamp biomicroscope was employed to examine the anterior chamber.

### 2.3 Electroretinography

Full-field ERGs were tested at 7–9 and 21–23 weeks of age using a LKC system (LKC Technologies, Gaithersburg, MD), as previously described (Liu et al., 2015). Briefly, the male and female rats were kept in the dark for a period of 2–8 h prior to ERG testing in order to enhance retinal sensitivity (Behn et al., 2003). The animals were anesthetized with a 2.0%–2.5% concentration of isoflurane in oxygen. A dim red light, generated by an Energizer red LED 315 headlamp (Intensity: ∼5 μW/cm^2^; wavelength: 620–645 nm; Energizer Holdings, Inc., MO), was briefly used to aid in animal manipulation and electrode placement. The body temperature was maintained using a heated pad connected to the ground. One drop of local anesthetic was administered to prevent blinking, and 1% tropicamide was applied to induce pupil dilation. ERG lens electrodes (Medical Workshop, Groningen, Holland) were placed on both eyes using artificial tears (GenTeal Tears, Alcon, Geneva, Switzerland) as a coupling agent. After disinfecting the skin with an alcohol pad, a platinum needle reference electrode (Natus Neurology, West Warwick, RI) was inserted subcutaneously between the eyes on the forehead. After scotopic testing, the animals were exposed to standard facility lighting (∼250 lux) for 10 min to allow for light adaptation prior to photopic ERG testing.

A UTAS BigShot Visual Electrodiagnostics System was used to evoke and acquire ERG signals that were high–pass filtered at 0.3 Hz and low–pass filtered at 500 Hz. ERG protocols were adapted from Rosolen et al. (2005) to test scotopic and photopic luminance responses of the retina. Photopic responses were obtained with the background Ganzfeld illumination of 30 cd/m^2^ (white light generated by the BigShot system and calibrated by LKC Inc.). ERG waveforms were analyzed using LKC Technologie’s software and the guidelines of the International Society for Clinical Electrophysiology of Vision (ISCEV) (Rosolen et al., 2005). The amplitude of the *a*-wave was measured from baseline to trough and its latency was measured from stimulus to *a*-wave trough. The amplitude of the *b*-wave was measured from *a*-wave trough to *b*-wave peak and its latency was measured from the stimulus to *b*-wave peak*.*

### 2.4 Optokinetic tracking response

Visual acuity was measured in the animals with normal (*n* = 9, male) and abnormal (*n* = 9, male) ERGs in group 3 (Table 1) using an optokinetic testing apparatus (OptoMotry; Cerebral Mechanics, Inc., Lethbridge, AB, Canada) at 21–23 weeks of age. It tested if the animal had reflexive head movement as the responses to rotating strips displayed on four computer monitors (optokinetic reflex) surrounding the animal (Chowers et al., 2017). A standard stepwise protocol was adapted, and the final score was calculated by the program, and the test videos were captured for post-experiment review and confirmation. Three observers’ judgments were pooled for determination of animal’s OKR responses.

### 2.5 Brainstem auditory evoked potential

Rats with normal (*n* = 9, male) and abnormal (*n* = 9, male) ERGs from group 3, used for OKR test, were also tested for BAEP at 21–23 weeks of age. The animals were anesthetized with 2.5% isoflurane and placed on a heated pad to maintain a body temperature of approximately 37.5°C. Acoustic stimuli were created using a digital stimulator (WPI DS8000, World Precision Instruments, Sarasota, FL) in the form of click stimuli with a 100 μs duration and a monopolar waveform. The stimuli (75 dB) were delivered bilaterally to the rat’s external auditory canals via earplugs. Six hundred and fifty stimuli were administered at a 5 Hz frequency. Auditory potentials were recorded from the right ear only through a subcutaneous Grass^®^ platinum needle electrode (F-E2, Natus Neurology, Galway, Ireland) placed at the vertex (active) and parietal-occipital area ventrolateral to the right ear (Alvarado et al., 2012). The signals were amplified 10,000 times, bandpass filtered between 300 Hz and 3,000 kHz, and sent to an Axon Digitizer (1550B, Molecular Devices Corp, Sunnyvale, CA) for analog-to-digital conversion. The responses were averaged 650 times, and the averaged waveforms were analyzed within a 20 msec post-stimulus window. Clampfit software (Molecular Devices, ver. 10.6) was used for measurements and analysis of amplitude and latency of evoked auditory responses. The peak amplitudes and latencies of waves II, III, IV, and V were determined relative to the onset of the acoustic stimulus (Alvarado et al., 2012).

### 2.6 Ultrasonic vocalization

Rats with normal (*n* = 8, male) and abnormal (*n* = 8, male) ERGs from group 3, used for OKR and BAEP tests, were also tested for USV at 21–23 weeks of age. To reduce social isolation effects on USVs (Brudzynski and Ociepa, 1992), rats were pair-housed in 8 cages for 24-h continuous recording of USVs. In the test cage, an ultrasound microphone was inserted and fixed in the center of the short wall to capture USV signals emitted by the rats. The emissions were captured by the UltraSoundGate condenser ultrasonic microphone (CM16, Avisoft Bioacoustics, Berlin, Germany), which is sensitive to frequencies between 15 and 180 kHz and has a flat frequency response between 25 and 140 kHz (±6 dB). The microphone was connected to a computer via an UltraSoundGate IH8 (Avisoft Bioacoustics), and acoustic data were recorded by Avisoft Recorder software (version 2.95, Avisoft Bioacoustics), using a sampling rate of 250,000 Hz in 16-bit format and a recording range of 0–125 kHz (Hwang et al., 2022). Fifty and 22 KHz signals were analyzed off-line.

### 2.7 Histology

One to 3 weeks after behavioral testing (OKR, USV and BAEP tests), the 18 male rats were selected for necropsy and tissue collection. These animals were deeply anesthetized using isoflurane and then euthanized by exsanguination. The brains were rapidly and carefully removed, sliced in half coronally, and then fixed overnight in 4% neutral buffered formalin. The following day, the specimens were trimmed coronally at the level of the striatum, corpus callosum, and motor cortex, as well as at the level of the mid-cerebellum and medulla oblongata (levels 2 and 6, as described in (Bolon et al., 2013)). The two most rostral sections of each brain level were processed and embedded into the same paraffin block, and 5 μm sections were taken. The eyes were enucleated immediately after the brain was collected and fixed in Davidson’s fixative. The eyes were then processed into slides for microscopic evaluation. For each eye, a horizontal section was taken just below the optic nerve and at least five step sections were taken at 100 μm intervals, starting from below the optic nerve and proceeding toward the optic disk. All brain and eye sections were stained with hematoxylin and eosin (H&E) for microscopic evaluations.

### 2.8 Data analysis and statistics

For ERG data, a two-way analysis of variance (ANOVA) with repeated measures was performed to compare and assess the luminance responses to light flashes (Inamdar et al., 2022), using GraphPad Prism (Version 9.0.0, GraphPad Software, San Diego, CA). Student *t*-test was used to compare the differences in ERG, OKR, USV, and BAEP parameters between normal sighted animals and those with abnormal ERGs. Fisher exact test was used for rate or incidence comparison. The statistical significance of the comparisons was determined at a level of α = 0.05 (Liu et al., 2015).

## 3 Results

### 3.1 Abnormal ERG in male Wistar Han rats

**FIGURE 1:**
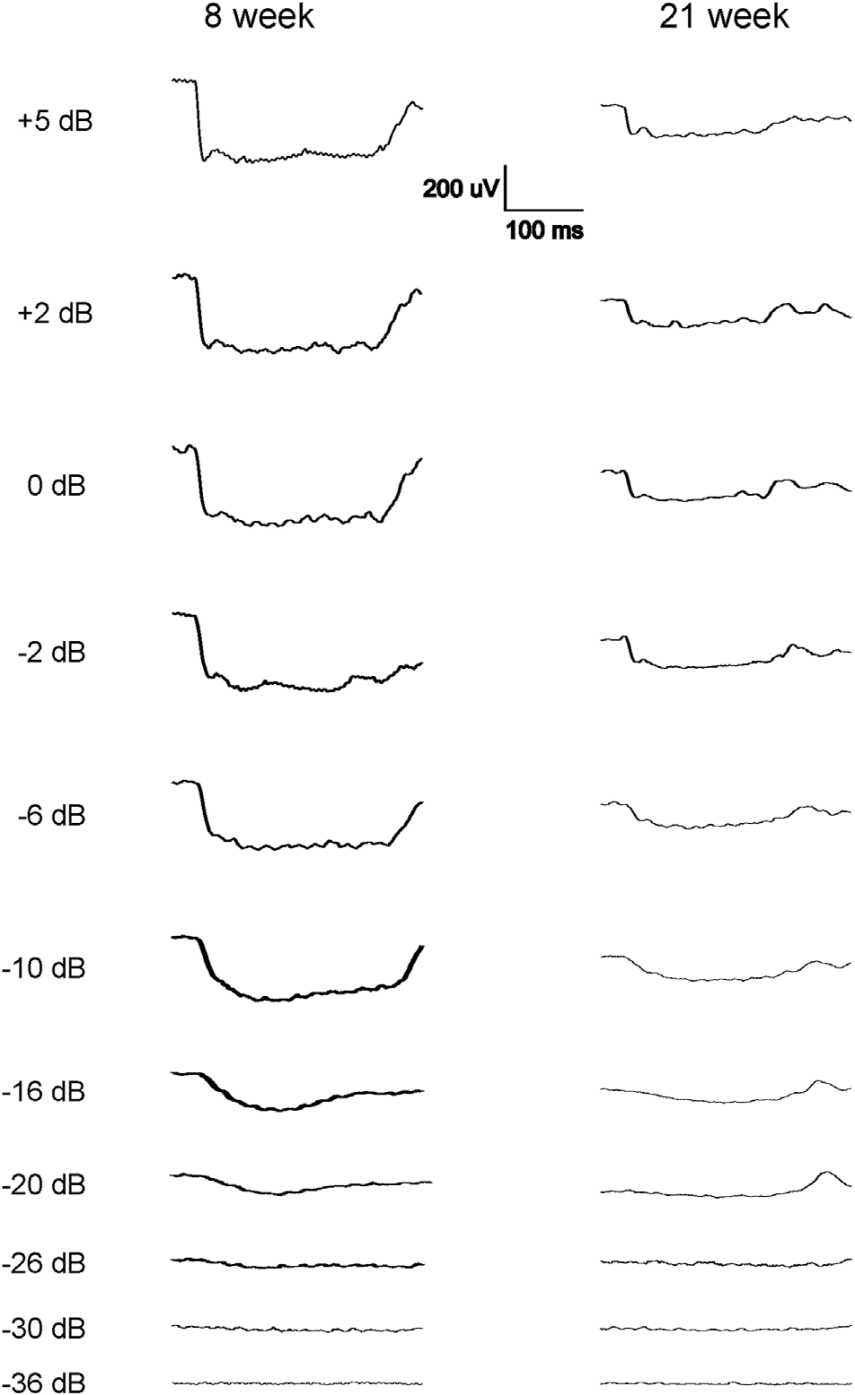
Representative abnormal scotopic ERG waveforms from some of the 7–9 weeks and 21–23-week-old male WH rats in response to a series of light flashes (−20 to 5 dB). Each waveform is the average of 3-9 responses to the same intensity flash. Following the flash stimulation of the eyes, no standard *a*-wave could be elicited, instead long (about 300 ms) negative waveforms were seen when the flash stimuli reached −20 dB, without further increase when the flashes were intensified. No standard *b*-wave was present in these animals.

**FIGURE 2:**
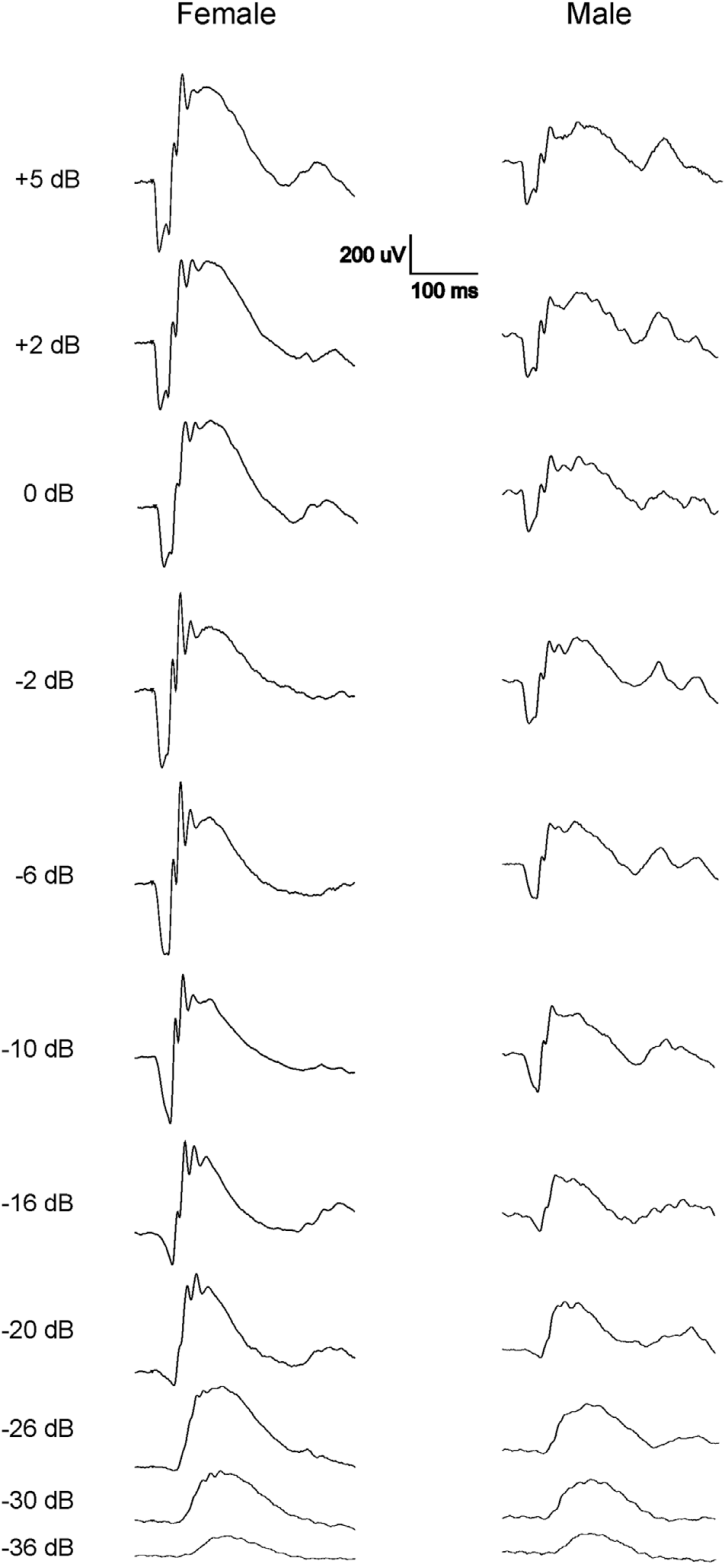
Representative normal dark-adapted ERG waveforms from 7-9-week-old female (left) and male (right) Wistar Han rats in response to flashes from −36 to 5 dB. Note that both *a*- and *b*-waves are larger in females compared with males at all tested flash levels.

The scotopic and photopic luminance responses to a series of flashes were tested in four groups of WH rats aged 7–9 and 21–23 weeks. For female animals, ERGs were tested at 2 ages within the same animals (group 2 and 4). Interestingly, some animals in both age groups displayed abnormal ERG waveforms, characterized by a large negative inflection followed by a flat line (Figure 1), without clear *a*- or *b*-waveform as seen in normalsighted animals (Figure 2). In addition, these waveforms did not increase in amplitude as the flash stimuli were intensified (Figure 1). Notably, this type of abnormal ERG waveform was only observed in males in groups 1 and 3 (13% and 19%, respectively), but not in age-matched females (0%, *p* = 0.0126, Fisher exact test, Table 1).

### 3.2 Normal ERG response comparison between male and female Wistar Han rats

**FIGURE 3:**
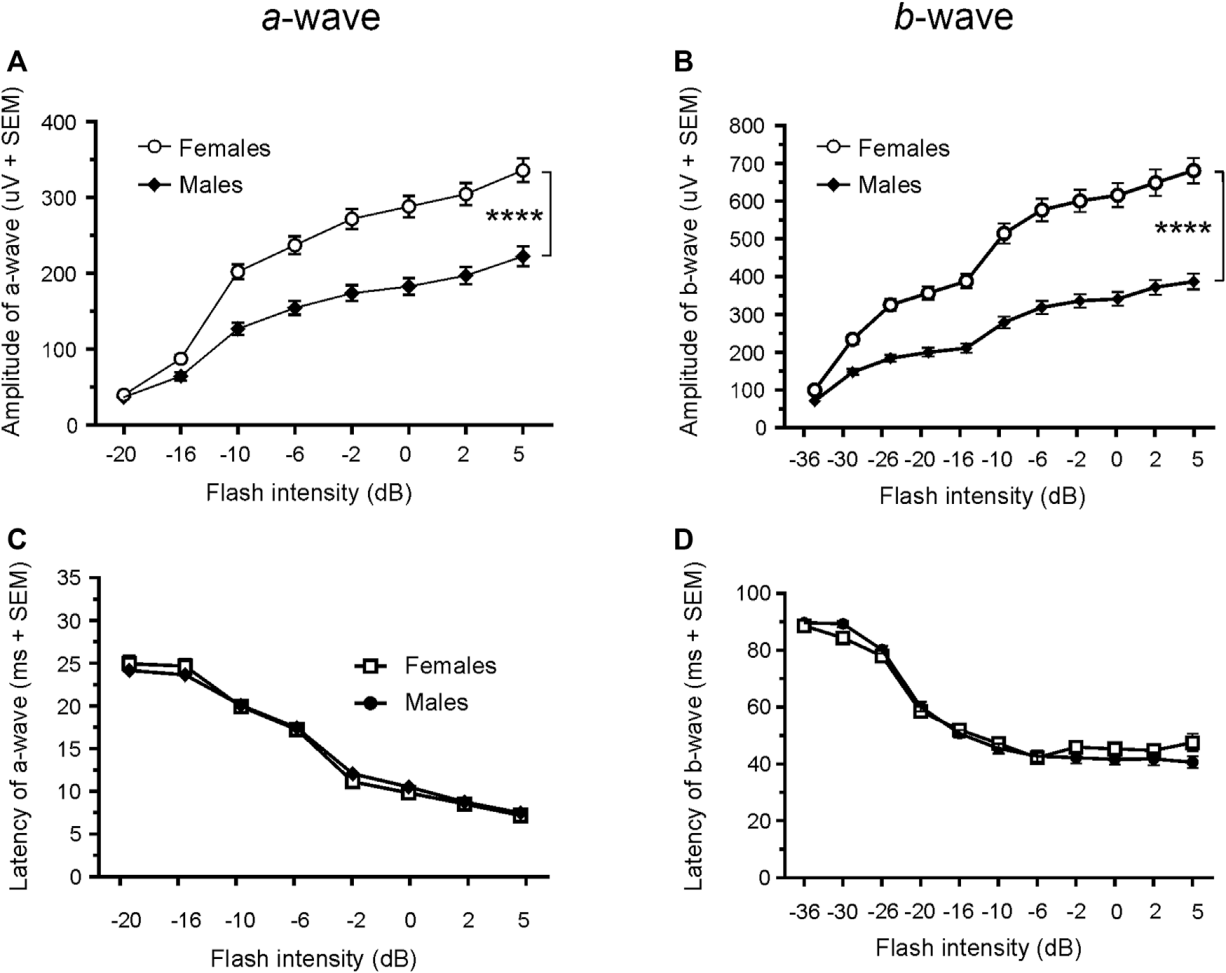
Comparison of scotopic *a*- and *b*-wave luminance responses between male and female WH rats with normal ERG signals at 7–9 weeks. Male Wistar Han rats had lower mean amplitudes of rod-mediated ERG *a*-wave **(A)** and *b*-waves **(B)** tested with −36 to +5 dB flashes that were statistically significant when compared with subset of female Wistar Han rats with normal ERG, but there were no statistically significant differences in the latencies of rod-mediated luminance response *a*- or *b*-waves **(C, D)** between the two groups of animals. SEM = standard error of the mean. * Indicates significant differences between male (filled circle) and females (open circle) at the same flash intensities of stimulation (2-way ANOVA, F (1,94) = 36.98, ****p < 0.0001 for **(A)** and F (1.94) = 56.02, *p* < 0.0001 for **(B)**.

**FIGURE 4:**
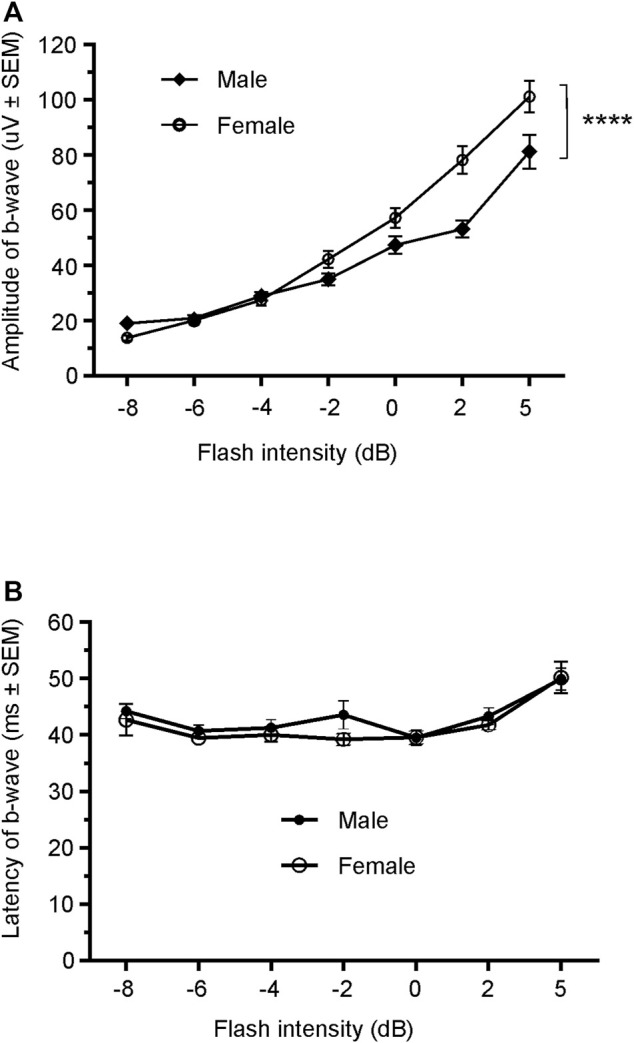
Comparison of photopic *b*-wave luminance responses between male and female Wistar Han rats with normal ERG signals at age of 7–9 weeks. Male Wistar Han rats had lower mean amplitudes of cone-mediated ERG *b*-wave **(A)** tested with −8 to +5 dB flashes that were statistically significant [2-way ANOVA, F (1,657) = 19.14, ****p < 0.0001] when compared with the female Wistar Han rats. But there were no statistically significant differences in the latencies of cone-mediated luminance response *b*-waves **(B)** between the two groups.

**FIGURE 5:**
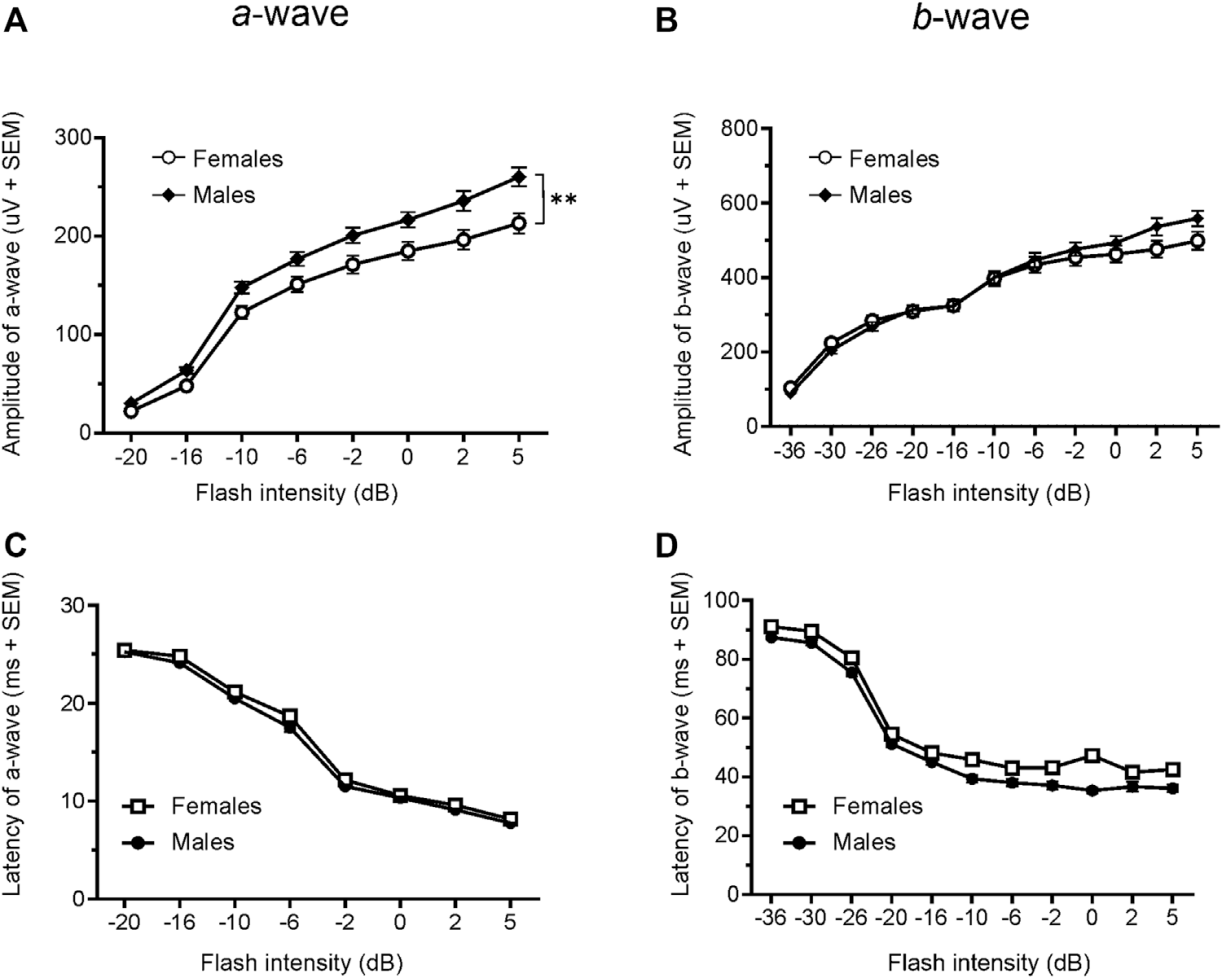
Comparison of scotopic *a*- and *b*-wave luminance responses between male and female Wistar Han rats with normal ERG signals at 21–23 weeks. Female Wistar Han rats had lower mean amplitudes of rod-mediated ERG *a*-wave **(A)** but not *b*-waves **(B)** tested with −36 to +5 dB flashes that were statistically significant [2-way ANOVA, F (1,88) = 8.210, **p = 0.0052] when compared with a subset of male Wistar Han rats. But there were no statistically significant differences in the latencies of rod-mediated luminance response *a*- or *b*-waves **(C, D)** between the two groups of animals.

**FIGURE 6:**
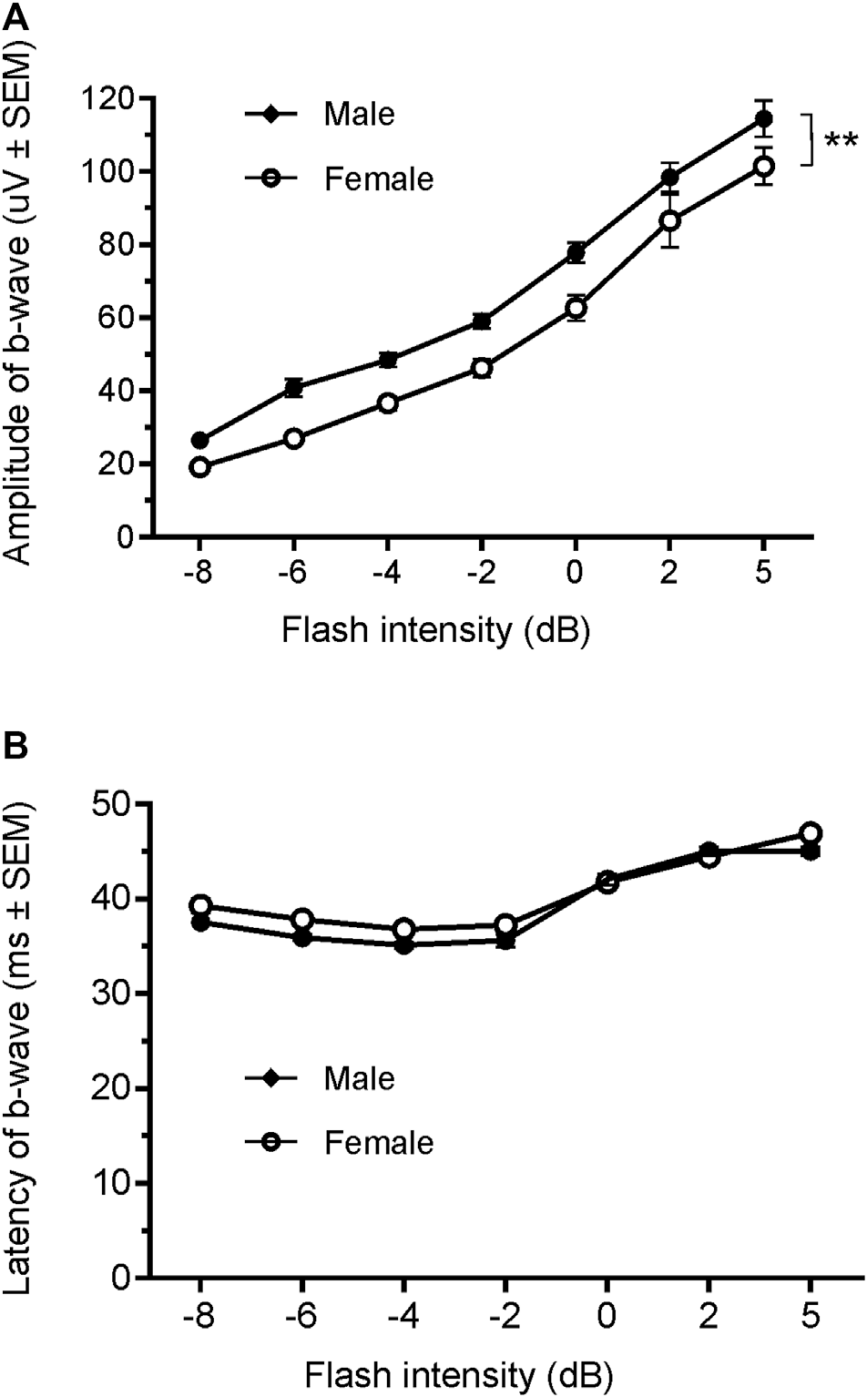
Comparison of photopic *b*-wave luminance responses between male and female Wistar Han rats with normal ERG signals at age of 21–23 weeks. Female Wistar Han rats had lower mean amplitudes of coned-mediated ERG *b*-wave **(A)** tested with −8 to +5 dB flashes that were statistically significant [2-way ANONA, F (1,88) = 11.21, **p = 0.0012] when compared with the male Wistar Han rats. But there were no statistically significant differences in the latencies of cone-mediated luminance response *b*-waves **(B)** between the two groups.

Since only male WH rats manifested abnormal ERG waveform, we wondered whether there were also any differences between males and females that had normal ERG responses. Therefore, we compared scotopic *a*-wave, *b*-wave, and photopic *b*-wave parameters between male and female animals at 7–9 weeks (45 males vs. 51 females) and 21–23 weeks (39 males vs. 51 females) of age. At 7–9 weeks, male WH rats (group 1) had lower mean amplitudes for rod-mediated scotopic ERG *a*-wave (Figures 2, 3A) and *b*-wave (Figures 2, 3B), which were statistically significant when compared with female WH rats. However, there were no differences in the latency of scotopic *a*- or *b*-waves between males and females (Figures 3C, D). The mean amplitudes of scotopic oscillatory potential were significantly lower in males compared to females, with a 43% difference (*p* < 0.0001, *t*-test). The male WH rats also had lower mean amplitudes for cone-mediated photopic *b*-wave (Figure 4A), but no differences in the latency of photopic *b*-waves between males and females (Figure 4B). At 21–23 weeks, in contrast to the comparative results obtained at weeks 7-9, male WH rats (group 3) had slight but significantly larger mean amplitudes (Figure 5A) and similar latency (Figure 5C) of *a*-wave, and similar *b*-wave amplitude and latency of rod-mediated scotopic ERG responses. Likewise, cone-mediated *b*-wave amplitude was larger in males compared with females (Figure 6A). There were no differences in the latency of *b*-weave ERG parameters tested at this time point (Figures 6B).

### 3.3 Normal ERG responses comparison between 7–9 and 21–23 weeks in female Wistar Han rats

Given the opposite difference of ERG responses between male and female animals at 7–9 and 21–23 weeks, we longitudinally compared the animals ERG responses in cohort 2 female animals (group 4 vs. group 2). Interestingly, the amplitudes of both scotopic ERG *a*- and *b*-wave, though not the latencies, were significantly decreased in 21–23 weeks compared with 7–9 weeks (*a*-wave: 213.0 µV vs. 335.9 µV at 5 dB; b-wave: 498.3 µV vs. 698.6 µV at 5 dB, all *p* < 0.0001, Figures 3A vs 5A and Figures 3B vs 5B).

### 3.4 Visual acuity in weeks 21–23

**FIGURE 7:**
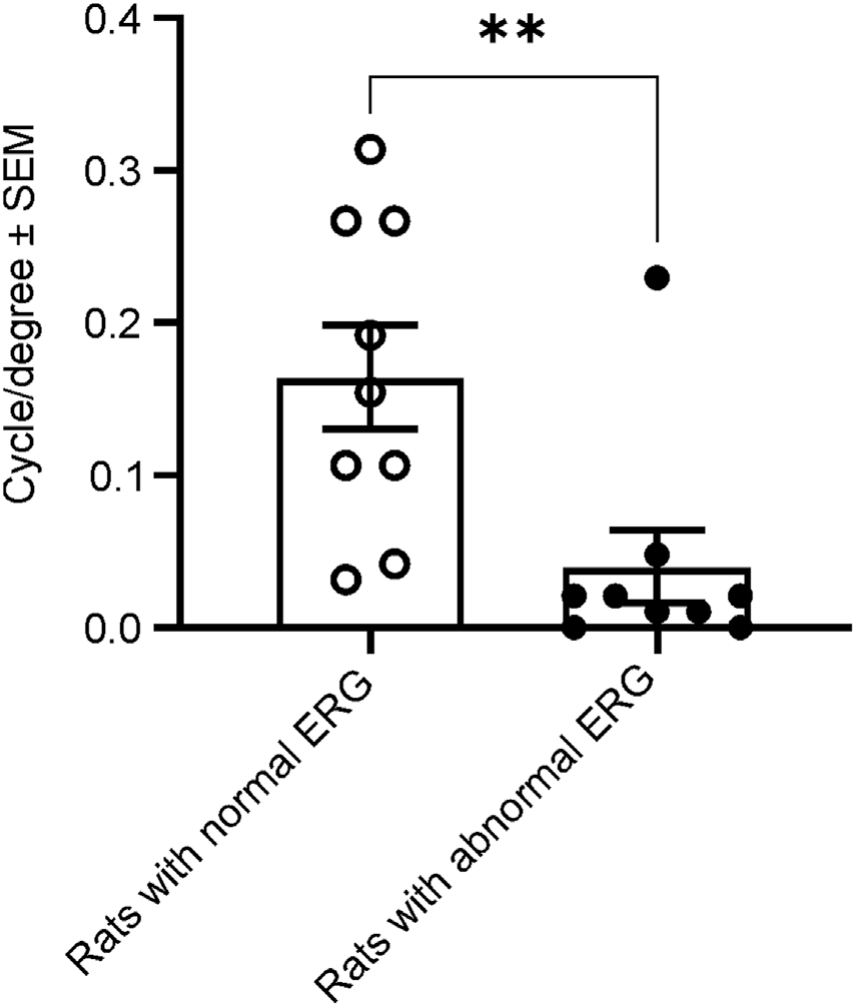
Comparison of spatial frequency thresholds of the acuity measured with OKR between animals with and without normal ERG waveforms at 21–23 weeks. The animals with abnormal ERG waveforms had significantly smaller grating thresholds (0.04 cycle/degree), compared with the animals with normal ERG (0.17 cycle/degree, *t*-test, t (Bavelier and Neville, 2002) = 2.978, **p = 0.0089).

To evaluate whether animals with abnormal ERG would exhibit normal visual-dependent behavior, we performed a visual acuity behavior test (OKR) in male rats at 21–23 weeks of age. We measured and compared visual acuity between 9 animals with normal and 9 animals with abnormal ERG waveforms. In the nine rats with normal ERG waveforms, the average visual acuity was 0.165 ± 0.102 cycle/degree (mean ± SD), whereas the mean visual acuity was only 0.040 ± 0.073 cycle/degree in 9 animals with abnormal ERG waveforms. Thus, the animals with abnormal ERG waveforms resulted in statistically significantly smaller mean visual acuity scores compared with the animals with normal ERG waveforms. (Figure 7).

### 3.5 BAEP in week 23

To determine if the animals with abnormal ERG have altered hearing function to compensate for poor vision, we measured and compared brainstem auditory-evoked potentials in the same group of animals tested for visual acuity (3.4). There were no statistically significant differences in the amplitudes or latencies of waves II, III, IV, and V between the animals with normal ERG and those with abnormal ERG waveforms (Supplementary Table S2; Supplementary Figure S1).

### 3.6 USV comparison in weeks 21–23

Ultrasonic vocalization, an important mean of communication between rats, was evaluated to investigate if there was compensation in USVs in the blind animals. We recorded the USV from 8 rats with normal and 8 with abnormal ERG previously used for BAEP test continuously for 24 h. The poor-sighted animals had similar circadian patterns and 24-h total USV counts as the normal-sighted animals in both 50-kHz and 22-kHz USV call counts (all *p* > 0.05, Supplementary Figure S2).

### 3.7 Clinical and ophthalmic observations

No signs of abnormal behavior or morbidity were observed in any animal throughout the 3-month period. The ophthalmological analyses revealed no abnormalities in the retina and other components of the eyes in group 2 animals (females) at 7–9 weeks of age or groups 1 and 3 animals (males) at 7–9 weeks of age and 21–23 weeks of age, respectively.

### 3.8 Histology

There were no abnormal microscopic findings in the retina, brainstem, and visual and auditory-related areas in the rats with abnormal ERG responses at 21–23 weeks of age (Supplementary Table S3).

## 4 Discussion

In this study, we evaluated retinal function in male and female WH rats at two ages to determine the presence of spontaneous retinal functional deficits in the albino strain, and to explore any potential compensations in other sensory systems. Our results showed that a fraction of male WH rats had abnormal ERG signals and poor visual-mediated tracking responses at both 7–9 weeks and 21–23 weeks of age, without any changes in retinal or brain morphology. Even in normal-sighted rats with normal ERG signals, we found that the scotopic and photopic luminance responses were smaller in male WH rats compared with age-matched females at 7–9 weeks, but not at 21–23 weeks. Here, we chose the ages to mimic the duration of regular 3-month sub-chronic toxicity studies (Galijatovic-Idrizbegovic et al., 2016), which usually starts at 5–9 weeks of age (Baldrick, 2008). We did not observe evidence of compensations in brainstem auditory potential, ultrasonic vocalizations, or auditory morphology in the visual pathway of blind rats at 21–23 weeks of age (to mimic the time point at which histopathology is routinely evaluated). In conclusion, these findings confirm the presence of spontaneous retinal ERG deficits in 13% of adult male WH rats at 7–9 weeks of age and ERG and OKR deficits in 19% of adult male WH rats at 21–23 weeks of age, respectively.

The most notable outcome of our study is that a subset of naïve male WH rats showed abnormal ERG responses when their eyes were stimulated with flashes. As depicted in Figure 1, the amplitude of the scotopic ERG barely increased as the stimuli grew brighter, a phenomenon similar to the waveforms reported previously in 8.5-week-old albino rats with retinal dystrophy (Dowling and Sidman, 1962). The missing amplitudes of both *a*- and *b*-waves in these rats could be a result of weaker or no activity of photoreceptors, or minimal input from photoreceptors (Dowling and Sidman, 1962) into the post-photoreceptor circuits in the neuroretina, such as bipolar cells. In addition to the abnormal ERGs, we also evaluated the visual acuity of rats with and without normal ERG waveforms at 21–23 weeks of age. The sighted animals had an average acuity of 0.17 c/d, which is slightly lower than the values (0.36 c/d) reported for male WH rats at 7–9 weeks (Redfern et al., 2011). This difference might be due to observer bias. Despite this apparent decrease, the animals with abnormal ERGs had significantly lower acuity values, providing further evidence of vision impairment in this subset of male WH rats. Our data analysis of other animals with normal ERG waveforms, similar to the results reported for 8–26 week-old Sprague-Dawley rats (Chaychi et al., 2015), confirmed that the average ERG luminance responses in male WH rats were significantly smaller than those of females (Figures 2, 3) at 7–9 weeks old (*p* < 0.01), suggesting functional differences in photoreceptors, particularly the rod photoreceptors. Interestingly, our microscopic evaluations showed no noticeable thinning or reduction of the photoreceptor nuclei layer in 21–23 weeks old male rats with abnormal ERGs compared to those with normal ERGs. This is consistent with a recent review paper, which found retinal degeneration in control WH rats only after 52- and 104-week toxicity studies (Cloup et al., 2021). Likewise, the routine ophthalmic examination did not find any abnormality in the eyes of male and female WH rats at 7–9 weeks of age or male HW rats at 21–23 weeks of age. For the female animals in group 4, no ophthalmic examination was repeated at 21–23 weeks of age, since the ophthalmic examination is less sensitive compared with histopathology or ERG in spontaneous (Taradach et al., 1981), light-induced (Jaadane et al., 2015) or systemically administered drug-induced (Huang et al., 2015) retinal damages. We hypothesized that visual functional impairment occurs before any morphological changes can be seen in these animals. We also don’t attribute the current observation to well-documented light-induced retinal damage often seen in albino rats [see review (De Vera Mudry et al., 2013)], since in our vivarium environment, 12 h on/12 h off cyclic illumination was applied during all the study course, which is less damaging to the retina than constant illumination (De Vera Mudry et al., 2013). We and animal vendor also used ∼300 lux lighting 1 m above the floor (Supplementary Table S4), which was approved safe and no phototoxic retinopathy concern for rats (Bellhorn, 1980). Furthermore, the animal cages were rotated vertically in the rack on a weekly basis as suggested (Rao, 1991). Rather, it may be inherited and related to albinism. It is well established that albino rats, such as Sprague-Dawley and WH, have impaired visual acuity (Prusky et al., 2002) and altered visual signal transmission latency from the retina to the superior colliculus (Thomas et al., 2005) compared with pigmented strains. These investigators did not further differentiate between sex, though. In humans, the influence of biological sex on retinal function as measured with the ERG has been known for over 60 years (Karpe et al., 1950). ERGs are typically reported to have larger amplitudes in women compared to men (Birch and Anderson, 1992; Brule et al., 2007). Estrogens have been demonstrated to be neuroprotective against a variety of insults in both *in vitro* and *in vivo* models of neurodegenerative diseases. It is believed that the differences in retinal function and structure between the sexes may be governed by differences in sex hormone profiles. The presence of estrogen receptors mRNA (Wickham et al., 2000) and protein (Kobayashi et al., 1998) in various layers of the rat retina (Kobayashi et al., 1998; Kumar et al., 2008) suggests that this hormone plays an important role in maintaining normal retinal function in females (Yamashita et al., 2010; Yamashita et al., 2011). Additionally, the menstrual cycle and accompanying hormonal fluctuations, particularly estrogen, have been observed to potentially modulate several ocular structures, including the retina in humans (Barris et al., 1980; Bassi and Powers, 1986). Preclinical experiments demonstrated that estrogen protects against postischemic tissue damage in rat retina (Nonaka et al., 2000), and glutamate-induced cytotoxicity in the retinal photoreceptor cells (Nixon and Simpkins, 2012) and ganglion cells (Kumar et al., 2005). In a light-induced photoreceptor degeneration rodent model, estrogen reduced rod and cone photoreceptor cell damage functionally and structurally (ARVO Annual Meeting Abstracts, March 2012). Other sex-dependent differences, such as retinal pigment epithelia or neurotransmitters (glutamate and GABA (Blaszczyk et al., 2004)) in the retina might play a role in our observation, but none of them has been compared between retinas of male and female albino rats.

The next intriguing question is how blind animals handle communication and orientation without the use of their major sensory function. In other words, whether or not the blinded animals had altered sensory functions as compensation. To answer these questions, we recorded USVs continuously for 24 h, and the animals with abnormal ERGs appeared to have similar circadian patterns to those with normal ERGs in both 50-kHz and 22-kHz call counts. The data suggest that in these blind rats, the eye may still retain its ability to detect light cues for coordinating circadian rhythms, similar to blind mole-rats (Hetling et al., 2005). However, it was not known if there was compensation in other sensory channels, such as USV or auditory function. According to our 24-h recording, the spontaneous USV call count per 30 min and total count of 50-kHz (Schwarting, 2018) and 22-kHz (Simola, 2015) over 24 h didn’t show any significant difference between these animals and other normal-sighted animals as groups. For BAEPs to click stimuli, the sources of waves I, II, III, IV, and V of the potential are the cochlear nerve, cochlear nuclei, superior complex, dorsal and rostral olive extrusion, and lateral lemniscus, respectively (Shaw, 1988; Chen and Chen, 1991). BAEP increase during the postnatal period and are sensitive to brainstem lesions such as tumors, trauma, hemorrhage, ischemia and demyelination (Legatt, 2002). Our results indicate the auditory function in the brainstem level of the animals with abnormal retinal or visual function appears the same as those in the normal-sighted animals. This study is the first to investigate compensatory mechanisms of WH rats with impaired vision. We did not observe compensatory responses in USVs and BAEPs as well as the histology of auditory and visual pathway in these animals. Further studies need to be performed to explore additional systems or functions potentially altered in these animals. The mechanism underlying the retinal functional differences and potential compensation remains to be elucidated in further studies. Transcriptomic analysis might provide more details (e.g., immune response, inflammation, apoptosis, Ca2+ homeostasis or oxidative stress (Kozhevnikova et al., 2013). Other sensory modalities, for example, the olfactory function, which has been found age-related (Kraemer and Apfelbach, 2004), might be worth exploring for possible sensory compensation in blind rats.

In conclusion, our study shows 13%–19% incidence of retinal functional deficits in naive males WH rats at 7–23 weeks of age. Therefore, sex differences should be considered when using Wistar Han rats in toxicity and safety pharmacology studies with regard to data interpretation of retinal functional assessments. In addition, pigmented rats, such as Long-Evans rats with less spontaneous (Heiduschka and Schraermeyer, 2008) or light-induced (Wasowicz et al., 2002) visual impairments, could be considered for stand-alone retinal toxicity tests (Heiduschka and Schraermeyer, 2008; Perlman, 2009; Liu et al., 2015; Shibuya et al., 2015), although it is not a standard toxicity study strain and has less information available for other non-ocular tissues. Pre-screening the male WH rats in the pre-dose phase of the planned toxicity studies with ERG endpoint is also recommended.

## Data Availability

The original contributions presented in the study are included in the article/Supplementary Material, further inquiries can be directed to the corresponding author.

## Supplementary material

Supplementary Figure 1 Representative waveforms of brainstem auditory evoked potentials (BAEPs) in a rat with normal ERG signals (solid trace) and an animal with abnormal ERG waveforms (dotted trace). All four characteristic waves of the rat BAEP (II, III, IV, and V) were comparable between the two groups (Unpaired t-test, t(16) = 0.2437–0.9041, all p > 0.05).

Supplementary Figure 2 Comparison of USV calls between animals with and without normal ERG waveforms. The two groups of animals had similar circadian patterns of 50-kHz **(A)** and 22-kHz **(B)**. Each data point is the total count of 30 min recording. There are no significant differences in the total count of 50-kHz **(C)** or 22-kHz **(D)** USV calls during the 24-hour recording course. (P >0.05, Unpaired t-test, t(6) = 0.2503, p = 0.8107 for 50 kHz, and t(6) = 1.691, p = 1.6910 for 22 kHz). Filled bar = light off; open bar = light on.
