# Supplementary material for: Sex-related differences in retinal function in Wistar rats: implications for toxicity and safety studies
Source: Front Toxicol. 2023 May 23;5:1176665. doi: 10.3389/ftox.2023.1176665 (PMC10259507; doi:10.3389/ftox.2023.1176665)
Supplement: Supplementary file 1 [file Table1.docx]

**Table S1 Animal ordering and use information**

| **Group** | **Receipt month and year** | **Receipt age (week)** | **Duration at Pfizer prior to tests (week)** | **Age for tests**  **(week)** | **Sex** |
| --- | --- | --- | --- | --- | --- |
| 1 | Jan, 2021 | 7-9 | 1-2 | 7-9 | Male |
| 2 | Jun, 2021 | 6-8 | 1-2 | 7-9 | Female |
| 3 | Nov, 2021 | 9-10 | ~11 | 21-23 | Male |
| 4 | Same as group 2 | | ~14 | 21-23 | Same as group 2 |
