# Supplementary figures and images for: Sex-related differences in retinal function in Wistar rats: implications for toxicity and safety studies
Source: Front Toxicol. 2023 May 23;5:1176665. doi: 10.3389/ftox.2023.1176665 (PMC10259507; doi:10.3389/ftox.2023.1176665)

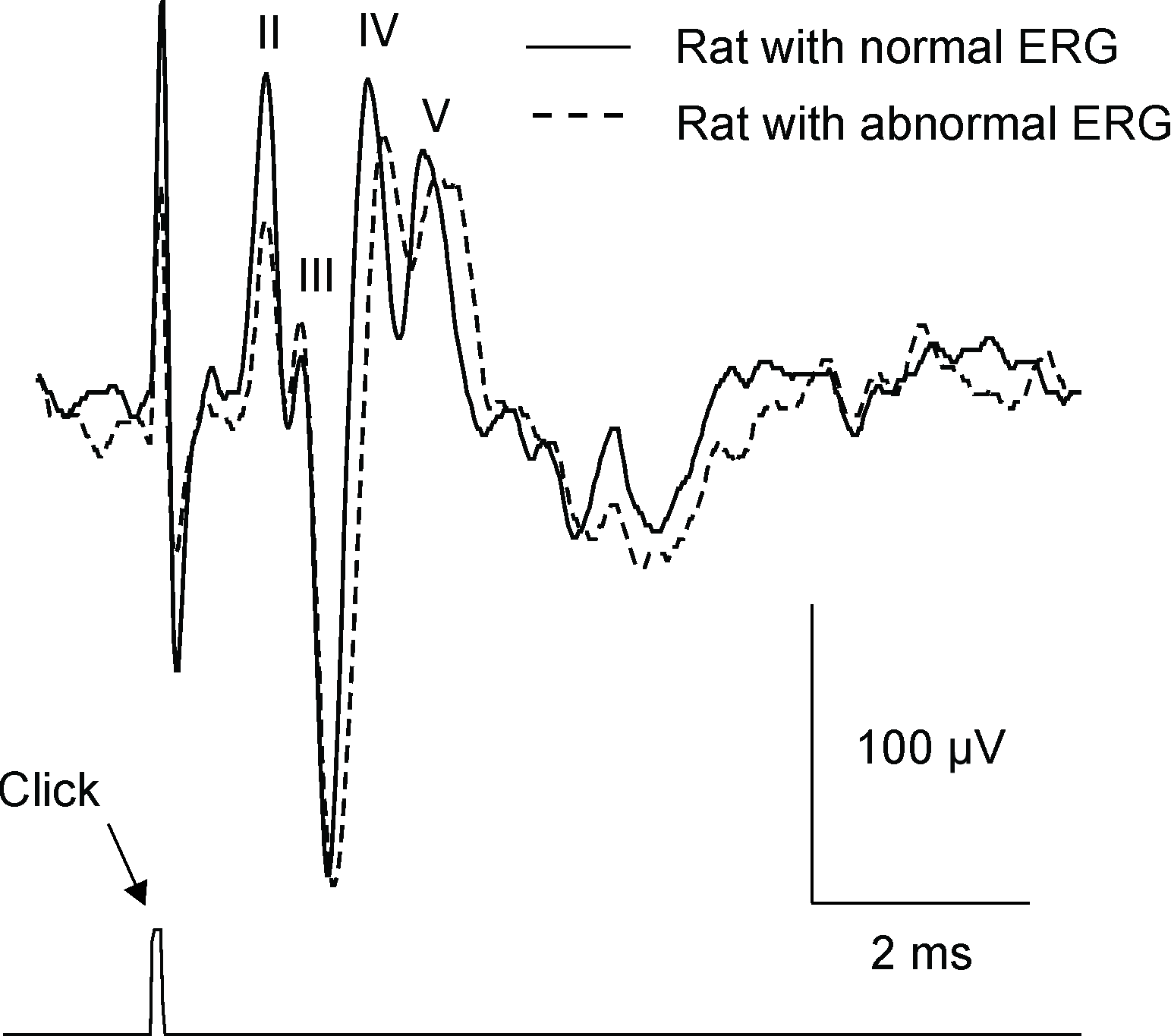

Supplement: Supplementary file 3 [file Image1.tif]
