# Supplementary material for: Sex-related differences in retinal function in Wistar rats: implications for toxicity and safety studies
Source: Front Toxicol. 2023 May 23;5:1176665. doi: 10.3389/ftox.2023.1176665 (PMC10259507; doi:10.3389/ftox.2023.1176665)
Supplement: Supplementary file 4 [file Table2.docx]

**Table S2. BAEP comparison between animals with normal ERG and abnormal ERG**

| **Waveform** | **II** | | **III** | | **IV** | | **V** | |
| --- | --- | --- | --- | --- | --- | --- | --- | --- |
|  | **Latency**  **(ms)** | **Amplitude**  **(µV)** | **Latency**  **(ms)** | **Amplitude**  **(µV)** | **Latency**  **(ms)** | **Amplitude**  **(µV)** | **Latency**  **(ms)** | **Amplitude**  **(µV)** |
| **Rats with normal ERG** | 1.06 ± 0.03 | 167.0 ± 51.3 | 1.40 ± 0.04 | 117.3 ± 36.5 | 2.10 ± 0.05 | 159.7 ± 52.4 | 2.61 ± 0.17 | 164.9 ± 40.6 |
| **Rats with abnormal ERG** | 1.12 ± 0.06 | 158.9 ± 45.1 | 1.46 ± 0.11 | 123.0 ± 57.0 | 2.20 ± 0.20 | 181.0 ± 66.5 | 2.72 ± 0.13 | 185.7 ± 55.8 |
| **P value*** | 0.014 | 0.726 | 0.138 | 0.808 | 0.185 | 0.461 | 0.135 | 0.379 |

Values are mean ± standard deviation. *: unpaired Student *t-*test
