# Supplementary material for: Sex-related differences in retinal function in Wistar rats: implications for toxicity and safety studies
Source: Front Toxicol. 2023 May 23;5:1176665. doi: 10.3389/ftox.2023.1176665 (PMC10259507; doi:10.3389/ftox.2023.1176665)
Supplement: Supplementary file 5 [file Table3.docx]

**Table S3. Histology summary**

| **Group** | **Age (week)** | **Sex** | **ERG** | **Animal tissue examined** | **Microscopic evaluation (H&E)** | |
| --- | --- | --- | --- | --- | --- | --- |
|  |  |  |  |  | **Eyes** | **Brain** |
| 3 | 21-23 | male | normal | 9 | within normal limits | within normal limits |
|  |  |  | abnormal | 9 | within normal limits | within normal limits |
