# Supplementary material for: Sex-related differences in retinal function in Wistar rats: implications for toxicity and safety studies
Source: Front Toxicol. 2023 May 23;5:1176665. doi: 10.3389/ftox.2023.1176665 (PMC10259507; doi:10.3389/ftox.2023.1176665)
Supplement: Supplementary file 6 [file Table4.docx]

**Table S4 Comparison of animal room condition between Pfizer and Charles River laboratory**

| **Vivarium** | **Light**  **(lux)** | **Temperature**  **(ºF)** | **Humidity**  **(%)** | **Air change rate**  **(/hour)** |
| --- | --- | --- | --- | --- |
| Charles River Laboratory, Raleigh, NC | 325* | 68-72 | 30-70 | 10+ |
| Pfizer, Groton, CT | 250-300 | 68-78.8 | 30-70 | 10+ |

*Measured 1 meter above the light blue floor
